# An improved pyrite pretreatment protocol for kinetic and isotopic studies

**DOI:** 10.1186/s12932-014-0010-0

**Published:** 2014-08-12

**Authors:** Natella Mirzoyan, Alexey Kamyshny, Itay Halevy

**Affiliations:** 1 Earth and Planetary Sciences, Weizmann Institute of Science, Rehovot, 76100 Israel; 2 Dept. of Geological and Environmental Sciences, Faculty of Natural Sciences, Ben-Gurion University of the Negev, Beer Sheva, 84105 Israel

**Keywords:** Pyrite oxidation, Elemental sulfur, Grain morphology, Etch pits, Sulfur isotopes

## Abstract

**Background:**

Pyrite is one of the most abundant and widespread of the sulfide minerals with a central role in biogeochemical cycles of iron and sulfur. Due to its diverse roles in the natural and anthropogenic sulfur cycle, pyrite has been extensively studied in various experimental investigations of the kinetics of its dissolution and oxidation, the isotopic fractionations associated with these reactions, the microbiological processes involved, and the effects of pyrite on human health. Elemental sulfur (S^0^) is a common product of incomplete pyrite oxidation. Preexisting S^0^ impurities as unaccounted reaction products are a source of experimental uncertainty, as are adhered fine grains of pyrite and its oxidation products. Removal of these impurities is, therefore, desirable.

A robust standardized pretreatment protocol for removal of fine particles and oxidation impurities from pyrite is lacking. Here we describe a protocol for S^0^ and fine particle removal from the surface of pyrite by rinsing in acid followed by repeated ultrasonication with warm acetone.

**Results:**

Our data demonstrate the presence of large fractions of S^0^ on untreated pyrite particle surfaces, of which only up to 60% was removed by a commonly used pretreatment method described by Moses *et al*. (GCA 51:1561-1571, 1987). In comparison, after pretreatment by the protocol proposed here, approximately 98% S^0^ removal efficiency was achieved. Additionally, the new procedure was more efficient at removal of fine particles of adhered pyrite and its oxidation products and did not appear to affect the particle size distribution, the specific surface area, or the properties of grain surfaces.

**Conclusions:**

The suggested pyrite pretreatment protocol is more efficient in removal of impurities from pyrite grains, and provides multiple advantages for both kinetic and isotopic investigations of pyrite transformations under various environmental conditions.

**Electronic supplementary material:**

The online version of this article (doi:10.1186/s12932-014-0010-0) contains supplementary material, which is available to authorized users.

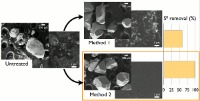

## Background

Pyrite (FeS_2_) is one of the most abundant and widespread of the sulfide minerals with a central role in biogeochemical cycles of iron and sulfur. Sedimentary pyrite formation and burial accounts for one to two thirds of the sulfur removed from the ocean [1]-[3], whereas pyrite weathering on land accounts for a similar fraction of the riverine flux of sulfate to the ocean [3]-[5]. The dissolution and oxidation of pyrite and other sulfide minerals in natural and anthropogenic environments exerts an important control on local environmental conditions, including pH and toxin chemistry [6]-[8]. Pyrite serves as an electron donor in some microbial metabolisms [9], and is often the final repository for sulfide generated during dissimilatory sulfate reduction [1], defining a role for pyrite in recording the isotopic history of Earth’s sulfur cycle.

Due to its diverse roles in the natural and anthropogenic sulfur cycle, pyrite has been extensively studied in various experimental investigations of the kinetics of its dissolution and oxidation [9]-[16], the isotopic fractionations associated with these reactions [17]-[22], the microbiological processes involved [23]-[25], and the effect of pyrite surface reactivity and hydroxyl radical formation on human health [26]-[29]. Common to many of these studies is the pretreatment of pyrite to prevent experimental artifacts and inaccuracies.

Mechanical breaking of pyrite to produce smaller grains creates dangling bonds [24], which result in increased oxidation rates by dissolved oxygen [10]. The presence of these dangling bonds promotes the formation of hydroxyl radicals [26], which are thought to be related to rapid production of sulfate upon exposure of pyrite to anoxic solutions (e.g., [26],[30]). In addition to the dangling bonds, crushing produces a fine coating of damaged, strained, mineral powder with high surface area, which adheres to grain surfaces, and initially elevates rates of oxidation, dissolution and reaction associated with pyrite surfaces (e.g., [11]).

Chemical alteration processes unaccounted for may also bias the outcome of experiments. Ferric iron oxide-like patches on the surface of pyrite grains are the first signs of oxidation of pyrite exposed to moist air [31]. These patches facilitate electron transfer between molecular oxygen and pyrite, thereby accelerating the oxidative dissolution of pyrite [32]. Longer exposure to moist air typically results in the wetting of the pyrite surface by formation of Fe-H_2_SO_4_ solutions, and ultimately the precipitation of Fe^3+^ or mixed-valence Fe^3+^/Fe^2+^ sulfates [15],[33]. Adhered patches of elemental sulfur (S^0^), present as a cyclic octaatomic solid (S_8_), are a common product of incomplete pyrite oxidation [34]-[36], and aqueous S^0^ is a known product of pyrite oxidation under acidic aqueous conditions [16]. Both iron sulfates and S_8_ may decrease the reactive surface area of pyrite and bias reaction rates downwards. Preexisting impurities in the form of Fe-H_2_SO_4_ solutions, Fe^3+^/Fe^2+^ sulfate minerals or S_8_ may additionally affect the speciation, concentration and isotopic composition of pyrite reaction products observed in experiments.

Of special concern is the effect of the above processes on experiments in which low-yield reaction products are of interest, such as determination of reaction kinetics and isotopic fractionations during pyrite oxidation reactions. In such experiments, anomalously fast initial rates, partially passivated surfaces, or preexisting impurities that are also expected reaction products may significantly affect the experimental results.

To minimize experimental biases due to physical and chemical alteration of the pyrite surface, pretreatment of pyrite grains is often practiced. Three common steps, all directed at the removal of oxidation products and adhering fine mineral grains are: i) rinsing with acid, ii) rinsing with an organic solvent, and iii) rinsing with distilled water. Rinsing with HF [11],[12], HCl [13],[18],[25] and HNO_3_[14] is sometimes combined with the use of organic solvents such as ethanol and acetone [11],[13],[25] to eliminate the oxidized surface layers and to remove adhered powders. Rinsing with an organic solvent is sometimes the only pretreatment procedure [15],[16]. Rinsing with water is described as a complementary step in several pyrite oxidation studies, aimed at removal of sulfate and other soluble sulfur oxyanions [11]-[13]. Although similarities exist among protocols, they vary in possibly significant ways. Some of the methods include only one or two of the described steps [16], whereas others include all three steps of pyrite pretreatment [12],[18]. Several authors [15],[16] have substituted prolonged rinsing [18],[20] with short-term ultrasonic treatment. The duration of all stages was largely variable, ranging from 1 minute to overnight soaking of pyrite samples in acid. In addition, the experimental focus should also guide the choice of pyrite pretreatment procedures. For example, acetone scavenges dangling bonds from Si(001) surfaces [37], and although its effects on pyrite surfaces have not been studied, the use of acetone and other organic solvents is not recommended when radical formation is desired, as in the study of the effects of pyrite on human health.

While numerous pyrite-cleaning methods have been used in experiments, a common pyrite pretreatment method, often used to investigate pyrite chemistry by the isotopic fractionations associated with it [18],[20]-[22],[38], is based on a protocol suggested by Moses *et al*. [30]. This method includes several rinses by HCl, acetone and deionized water. In addition to the putative removal of oxidized phases, the popularity of this method is based on the relatively high extraction efficiency of surface-bound sulfate during rinsing with water, and on the gentle effect of pretreatment agents on pyrite grain morphology. However, the efficiency of this procedure in extraction of S^0^, as well as other adhered oxidation products has not been assessed.

Here, we suggest an alternative pyrite pretreatment procedure, which removes impurities of S^0^, sulfate and other soluble oxyanions, adhered oxidation products and fine particles from crushed pyrite grains with high efficiency, while maintaining surface intactness and the original size distribution. We compare this procedure with the commonly used protocol reported in Moses *et al*. [30] and show that it is preferable in studies that are sensitive to small degrees of contamination by S^0^ and to adherence of very fine-grained materials.

### Experimental

Pyrite samples (Strem Chemicals, Newburyport, MA) were sieved to select a particle size fraction of 250 to 500 μm and pretreated by following methods:

Method 1 (according to Moses *et al*. [30]): Two batches of FeS_2_, 3 g each, were boiled in 50 mL of 6 M HCl for 15 minutes, washed with preliminarily de-oxygenated (by N_2_ purging for 2 hours) ultrapure deionized water (18.2 MΩ cm), rinsed twice with 50 mL of boiling 6 M HCl and three times with 50 mL of warm acetone.

Method 2: Two batches of FeS_2_, 10 g each, were boiled in 50 mL of 6 M HCl for 15 minutes, and washed with preliminarily de-oxygenated (by N_2_ purging for 2 hours) ultrapure deionized water (18.2 MΩ cm). Samples were cleaned in warm acetone in an ultrasonic bath with a frequency of 38 kHz (SW 6H, SonoSwiss). Twelve cycles of ultrasonication were performed, 15 minutes each. 100 mL of acetone was used in the first and second cycles, and 50 mL was used in the following 10 cycles. The greater volume of acetone used in the first cycles allows extraction of a larger amount of S^0^ before saturation is reached, at which point no additional S^0^ can be removed from the pyrite. Pretreated pyrite samples were dried in a desiccator under anaerobic conditions.

The organic solvents and HCl were not de-oxygenated in any of the procedures. The final extraction of S^0^ from samples pretreated by both methods, as well as untreated controls, was by addition of 50 ml of methanol to 1 g subsamples in an anaerobic glovebox, followed by gas-tight sealing of the samples and shaking overnight at room temperature (21°C) [39]. The S^0^ content was measured by high-performance liquid chromatography (HPLC, 1260 Infinity, Agilent Technologies). The relative standard error of HPLC injection was ~1%. The detection limit was 0.3 μmol l^-1^, the lowest measured concentrations were ~4 μmol l^-1^, and the relative standard error was ~2% due to background noise.

Pyrite grain surfaces were examined before and after cleaning by scanning electron microscopy (SEM, Zeiss Supra-55 VP, FEG). For this purpose, dried pyrite samples from the various stages of treatment were sprinkled on adhesive carbon tape mounted on aluminum stubs. The specific surface area of the pyrite was determined by 6-point BET (Quantachrome Instruments, USA) measurements using N_2_ gas after overnight degassing at 70°C. Particle size distributions were determined by a laser diffraction technique (Malvern Mastersizer 2000, Malvern Instruments, Worcester, UK). Each sample was transferred to a fluid module containing deionized water, and subjected to 2 consecutive measurements, each 3 minutes long.

## Results

### Elemental sulfur content

Approximately 1.8 μmol S^0^ g^-1^ FeS_2_ was extracted by methanol from untreated pyrite grains. When boiled in 6 M HCl for 15 minutes (Methods 1 and 2), the pyrite sample would rapidly turn the colorless acid to dark yellow, indicating the presence of a large amount of dissolved or suspended fine material (not quantified). During subsequent acid and acetone rinses in Method 1, no yellow color developed, but modest turbidity was observed.

**Figure 1:**
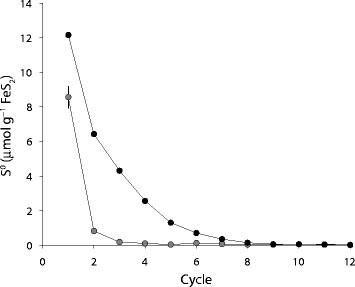
**Extracted elemental sulfur (S**^**0**^**).** The amount of elemental sulfur extracted by warm acetone from pyrite samples during 12 consecutive ultrasonication cycles (Method 2; see text for details). The gray and black circles represent two different batches of pyrite (10 g of batch 1, 30 g of batch 2). Error bars for batch 1 represent duplicate analyses and are smaller than the marker size where unseen. No duplicate analyses were made for batch 2, and instrument errors are smaller than the marker size.

High turbidity developed after the first stage of ultrasonic rinsing in acetone for 15 minutes in Method 2. Approximately 85% of S^0^ was also removed in this cycle (Figure 1), suggesting that some of the turbidity may be due to saturation of S^0^ and the formation of fine particles. Subsequent cycles yielded substantially smaller amounts of suspended materials, although minor jumps in turbidity were sometimes observed.

Overall, Method 2 was more efficient in removing S^0^ from pyrite grains. After pretreatment, approximately 98% S^0^ removal efficiency was achieved in the final methanol-extracted samples in Method 2, compared to 56-60% in Method 1. In a second experiment, pretreatment of 30 g of pyrite (different batch from the same supplier) by Method 2 resulted in more gradual S^0^ removal by acetone (Figure 1). However, after 8 ultrasonication cycles the amount of S^0^ in the acetone was similar to the amount extracted from the first batch of pyrite after the same number of cycles (Figure 1). Moreover, routine pretreatment procedures to clean pyrite grains for experiments in our laboratory result in similar final methanol-extracted S^0^ content in multiple samples, all ranging from 0.02 to 0.05 μmol S^0^ g^-1^ FeS_2_. This indicates that the treatment suggested here is robust to the specific source and storage history of the sample.

### Particle morphology, size distribution and surface area

Ground pyrite exhibits a wide range of sizes from very fine particles adhering to the surfaces of larger individual grains, to small particles scattered among the larger grains. The particle size distribution of crushed pyrite samples importantly affects the pyrite’s reactivity [11],[22], and variation in the relative abundance of the different size fractions, and/or the morphology of pyrite grains can significantly alter the outcomes of pyrite oxidation or dissolution experiments [40].

### Pyrite particle size distributions and specific surface area

**Figure 2:**
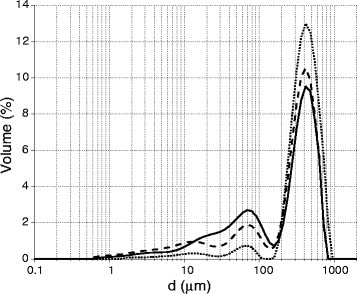
**Particle size distributions.** Size distributions of untreated pyrite (solid), and pyrite pretreated by Method 1 (dotted) and Method 2 (dashed).

**Figure 3:**
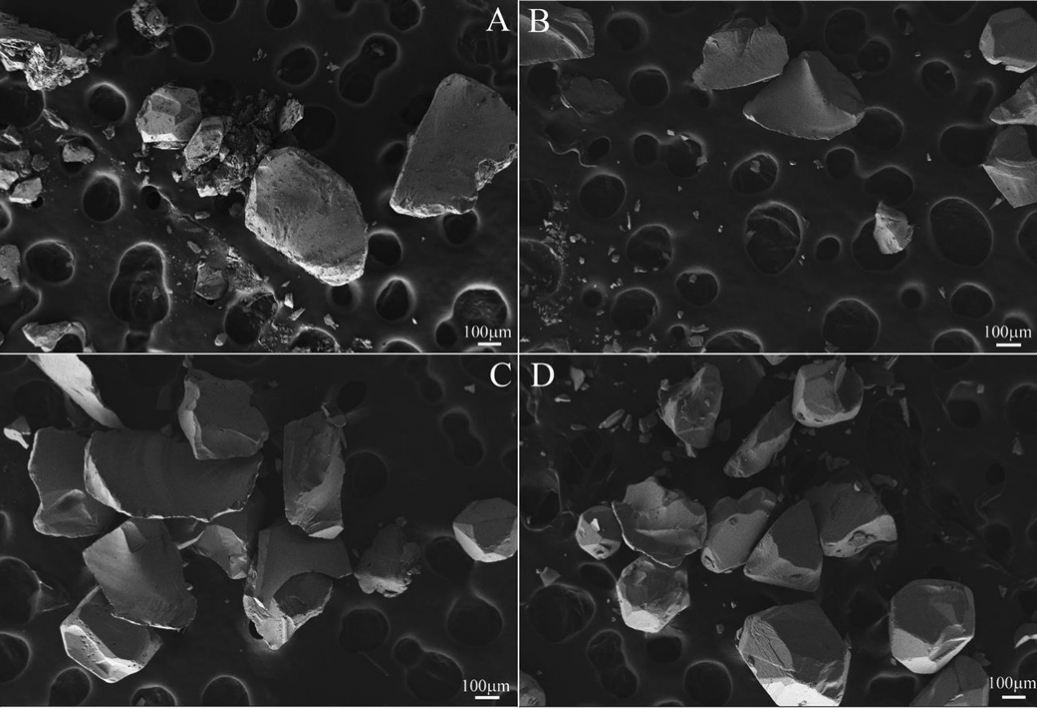
**Grain morphology observed by SEM.** Morphology of untreated pyrite **(A)**, pyrite after 15 minutes in boiling HCl **(B)**, and pyrite after pretreatment by Method 1 **(C)** and Method 2 **(D)** (see text for details).

Differences in particle size distributions between samples were small (Figure 2). Although all samples were sieved to achieve a relatively uniform size distribution of 250-500 μm, the actual particle sizes were more diverse, with a broad secondary peak at ~70 μm accompanying the main peak at ~400 μm, and a non-negligible fraction of grains with a size up to ~1000 μm. Pretreatment by both Methods 1 and 2 decreased the abundance of grains smaller than ~200 μm, but did not change the size distribution of the grains at the target range of sizes (Figures 2 and 3).

The specific surface area of untreated pyrite samples and samples pretreated by Method 1, as determined by BET measurements were identical, 0.03 m^2^ g^-1^ FeS_2_. Pyrite samples, pretreated by Method 2 displayed a specific surface area slightly greater than untreated samples (0.04 m^2^ g^-1^ FeS_2_), but within measurement error.

### Surface-attached particles

**Figure 4:**
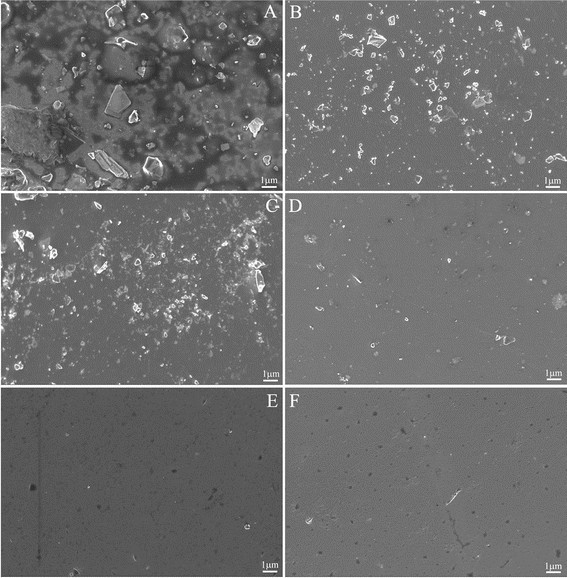
**Surface properties observed by SEM.** Surface of untreated pyrite **(A)**, pyrite after 15 minutes in boiling HCl **(B)**, pyrite after pretreatment by Method 1 **(C)**, and pyrite after the 3^rd^**(D)**, 6^th^**(E)** and 12^th^**(F)** ultrasonication cycle in Method 2 (see text for details).

The surface of untreated pyrite grains was covered in adhered mineral powder (Figure 4A). Large amounts of surface-attached particles were still observed after treatment by Method 1 (Figure 4C), indicating only partial removal of such particles by this method. Pretreatment by Method 2, however, resulted in almost complete removal of these fine particles already after the third cycle of ultrasonication (Figure 4D), and in much cleaner grain surfaces by the end of cleaning procedure (Figure 4F).

### Surface intactness

Oxidant attack on pyrite grains is non-uniform, occurring at specific sites of high excess surface energy, such as defects, solid inclusions, fluid inclusions, and some cleavage and fracture traces [19],[26]. The morphology of pyrite grains, therefore, affects the pyrite’s reactivity. The presence of corrosion pits and defects was checked visually (SEM) to assess the degree of weathering of the pyrite as a result of pretreatment. The surfaces of pyrite grains pretreated by Method 2 were covered by small pits (Figure 4F), whereas the surfaces of grains after pretreatment by Method 1 were covered in fine particulate matter and the identification of new or preexisting pits was not possible (Figure 4C).

## Discussion

The main objective of pyrite pretreatment procedures is the removal of preexisting impurities (e.g., oxidation products, adhered fine particles), which may bias experimental results or otherwise affect reaction rates. In the following discussion we evaluate the performance of the recommended protocol of pyrite pretreatment by multiple ultrasonication cycles using warm acetone as a solvent (Method 2), and compare this performance with the commonly used protocol developed by Moses *et al*. [30] (Method 1).While existing pyrite pretreatment methods putatively remove various soluble intermediate sulfur oxidation products by rinsing with water, as well as water-insoluble oxidation products (iron oxides and sulfates) by rinsing in HCl, the elimination of S^0^, reported to compose a relatively large portion of pyrite grain impurities [34]-[36], has not been addressed. Our data demonstrate the presence of large fractions of S^0^ on untreated pyrite particle surfaces, suggesting that S^0^ removal is a necessary step when investigating reactions involving pyrite. Furthermore, the existence of this S^0^ impurity in wholly untreated samples indicates that the S^0^ was not produced by boiling in HCl. Samples pretreated by both Method 1 and Method 2 contained less methanol-extracted S^0^ than untreated samples. However, Method 2 was found to be more effective at S^0^ removal than Method 1, and was capable of removing virtually all S^0^ from the pyrite grains (compared with up to 60% of the S^0^ removed by Method 1). Additionally, Method 2 was successful in treating approximately 3 times more pyrite with the same volumes of solvent used in Method 1, and can be advantageous if large amounts of pyrite need to be cleaned.

The development of dark yellow color in previously colorless acid during boiling in HCl, common to both methods, indicates the presence of dissolved or suspended fine material. This material probably comes from both the dissolution of iron sulfates, oxides and hydroxides, which can color solutions yellow, and the formation of colloidal S^0^ particles. Subsequent acid and acetone rinses in Method 1 resulted in the development of only modest turbidity, whereas high turbidity was observed in acetone during the first ultrasonication cycle in Method 2, indicating the removal of large amounts of fine materials in this method, compared to Method 1, as a result of vigorous agitation. Subsequent ultrasonication cycles in Method 2 episodically released additional fine particles, emphasizing the need for multiple ultrasonication cycles.

Iron oxide-like patches, which facilitate exchange of electrons between molecular oxygen and pyrite, thereby increasing rates of pyrite oxidative dissolution [32], as well as iron sulfates, which are common products of pyrite oxidation in air [15],[33], are presumably removed by rinsing in boiling HCl, common to Method 1 and Method 2. However, we observed a large number of bright particles, which appear to adhere to the pyrite surface even after rinsing in boiling HCl, and are only removed after multiple cycles of ultrasonication in warm acetone (compare Figure 4C and F). Visually, these precipitates appear very similar to iron sulfates observed by Jerz and Rimstidt (2004) [15], and it is possible that these particles are iron sulfates or oxides that were not fully dissolved in the HCl, and were only successfully dislodged during aggressive agitation by ultrasonication in Method 2. If this is the case, rinsing in boiling HCl for a duration longer than 15 minutes may be necessary to remove these particles chemically rather than mechanically. Alternatively, iron sulfates and oxides were removed by boiling HCl in both methods, and the observed particles were composed of S_8_, which was only successfully dissolved during ultrasonication in acetone in Method 2. Removal of S_8_ particles is desired, as they cover reactive pyrite surface area and may decrease apparent reaction rates. Whatever the identity of the adhered particles, it appears that Method 2 successfully removes them from the pyrite surface (Figure 4F).

Most pyrite transformation reactions depend on the surface area of exposed pyrite available for reaction. As such, the particle size distribution, the specific surface area, and the nature of the surface affect reaction rates measured in laboratory experiments. Differences among specific surface areas of untreated samples and those treated by Methods 1 and 2 (0.03, 0.03 and 0.04 m^2^ g^-1^ FeS_2_, respectively) are negligible. Pyrite grains sieved to a range between 250 and 500 μm display a size distribution wider than expected (Figure 2). The non-equant shape of the grains results in the occurrence of grains larger than the expected range (up to 1000 μm along certain dimensions; e.g., Figure 3C). The occurrence of grains distinctly smaller than the expected range is related to the adherence of fine particles to the larger grains, which are partly removed by both methods, but more effectively by Method 1 (Figure 2). The veracity of this minor difference between the methods is uncertain, given the small number of grain size measurements made here. However, breaking of larger particles to generate smaller particles by the more aggressive agitation in Method 2 can apparently be ruled out on the basis of the absence of change in the size distribution of the large particles (200-1000 μm), and by the shape of the secondary peak in the size distribution (10-100 μm; Figure 2). We conclude that neither method alters the size distribution in the desired range, and that both are moderately effective at removal of adhered fines.

The nature of the pyrite surface differs drastically among untreated samples, those treated by Method 1, and those treated by Method 2 (Figure 4). Both methods appear to remove a substantial fraction of the fine adhered particles during boiling in HCl (compare Figure 4A and B). However, any additional effect of Method 1 on the cleanliness of the surface is undetectable (compare Figure 4B and C), whereas Method 2 effectively scours the pyrite surface, removing almost all of the bright patches/particles observed after acid rinsing (compare Figure 4B and F). As discussed above, these particles are likely either iron sulfates or oxides, or S_8_, all of which can affect observed reaction rates in various ways.

Interestingly, the pyrite surface pretreated by Method 2 displayed pits up to ~400 nm in diameter, as well as elongated fractures. The number of pits increased with cycles of ultrasonication, concurrently with the decrease in the abundance of bright particles (Figure 4D-F). The origin of the observed pits is difficult to evaluate. They may be: i) generated by the repeated cycles of ultrasonication, or ii) initially present on the pyrite surface, but obscured by fine particles of pyrite and its oxidation products (iron sulfates, oxides and S_8_). In the latter case, the pits only become visible after successful removal of the obscuring particles. The apparently similar spatial distribution of adhered fine particles on pyrite grains treated only with HCl or by Method 1 and that of the exposed pits on grains treated by Method 2 (compare Figure 4B and F) supports the preexistence of the pits. We suggest two explanations for the apparent spatial association of the bright particles with the pits. The pits may protect adhered fine particles of pyrite and its oxidation products from removal by mechanical or chemical means, resulting in a preferential fine-particle residue in and around the cavities and requiring relatively aggressive mechanical agitation like the ultrasonication of Method 2. Alternatively, the bright particles are iron oxides, which facilitate electron transfer and elevate local rates of pyrite oxidative dissolution [32], resulting in the formation of dissolution pits in their immediate vicinity.

There are several advantages to the more efficient removal of S^0^ and fine particles in the protocol we suggest here. In kinetic pyrite oxidation experiments, reaction rates are calculated based on the removal of the reactant and/or production of sulfur oxides, mainly sulfate [18],[21]. Large uncertainties in reaction rate calculations are attributed to the efficiency of recovery of intermediate oxidation products and to pyrite surface area [21], among other factors. The ignorance of S^0^ impurities as a source of additional oxidation products during pyrite oxidation is a source of uncertainty. For example, an untreated impurity of 1.8 × 10^-3^ moles of S^0^ per gram of pyrite, as found in this study, would constitute 9.7% of the products generated from complete oxidation of the pyrite-S^0^ mixture. Partial oxidation of the pyrite-S^0^ mixture could potentially yield an even larger error, depending on the relative rates of oxidation of pyrite and S_8_. More rapid oxidation of the latter would result in its contribution to the oxidation products exceeding 9.7%. Further complications in characterizing the products of pyrite oxidation may arise in cases when oxidation of pyrite and S^0^ lead to the formation of sulfur-bearing compounds with different oxidation states, as suggested during anaerobic pyrite oxidation [6],[30],[41],[42]. If treated using the protocol suggested by Moses *et al*. [30], the remaining S^0^ impurity (~40% of the original impurity) would still constitute ~3.9% of the complete oxidation products. Pretreatment using the protocol recommended here would result in the remaining S^0^ impurity constituting only ~0.2% of the complete oxidation products.

An incompletely treated S^0^ contamination has implications also for the use of sulfur isotopes as probes of pyrite oxidation mechanisms. The isotopic composition of sulfur in the oxidation products reflects the isotopic composition of the sulfur source (in this case, pyrite and contaminant S^0^) and any isotopic fractionations associated with the reactions involved in pyrite oxidation [18]. As such, the isotopic composition of sulfoxy-anions produced from mixed oxidation of pyrite and contaminant S^0^ will reflect also the isotopic composition of the contaminant S^0^ and any isotopic fractionation associated with its oxidation, which in all likelihood differs from the fractionation associated with pyrite oxidation. For example, sulfate produced by abiotic oxidation of pyrite by O_2_ and Fe^3+^ is depleted in ^34^S relative to the pyrite by ~0.1‰ and ~0.7‰, respectively [18]. In an experiment of complete pyrite oxidation by Fe^3+^, an untreated S^0^ impurity of 1.8 × 10^-3^ mol g^-1^ FeS_2_, originating from oxidation by the O_2_ in air, would result in an apparent fractionation between the pyrite and the product sulfate of ~0.64‰ instead of ~0.7‰. However, in experiments aimed at determining fractionation factors, only a small fraction of the reactant is typically allowed to transform to the product to avoid isotope distillation. If, for example, only 2% of the pyrite was allowed to oxidize by Fe^3+^, but the same S^0^ impurity existed, the apparent fractionation would be only ~0.2‰. Such inaccuracies may be even more troublesome for the analysis of the minor sulfur isotopes, ^33^S and ^36^S.

## Conclusions

We have developed a novel protocol for elemental sulfur removal from pyrite surface by ultrasonication with warm acetone and compared this protocol with a commonly used technique [30]. The new procedure is more efficient at removal of S^0^, as well as fine particles of pyrite and its oxidation products adhered to the large pyrite grains. Furthermore, the procedure does not appear to adversely affect the particle size distribution, the specific surface area, or the properties of grain surfaces. Given these multiple advantages of the proposed pyrite pretreatment method, we recommend its use in both kinetic and isotopic investigations of pyrite transformations under various environmental conditions.

## Authors’ contributions

NM and IH designed the study and wrote the manuscript. NM performed the experiments and analyses. AK performed the S^0^ analyses. IH supervised the study. All authors have read and approved the final manuscript.

## Authors’ original submitted files for images

Below are the links to the authors’ original submitted files for images. Authors’ original file for figure 1 Authors’ original file for figure 2 Authors’ original file for figure 3 Authors’ original file for figure 4
