# Supplementary figures and images for: An improved pyrite pretreatment protocol for kinetic and isotopic studies
Source: Geochem Trans. 2014 Aug 12;15:10. doi: 10.1186/s12932-014-0010-0 (PMC4158268; doi:10.1186/s12932-014-0010-0)

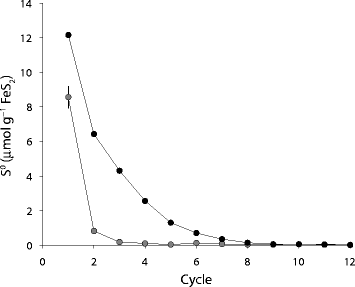

Supplement: Supplementary file 1 — Authors’ original file for figure 1 [file 12932_2014_10_MOESM1_ESM.gif]

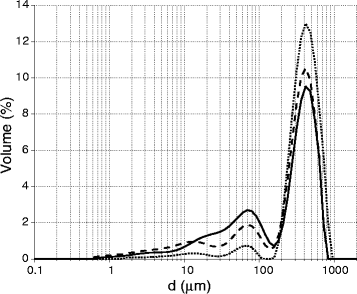

Supplement: Supplementary file 2 — Authors’ original file for figure 2 [file 12932_2014_10_MOESM2_ESM.gif]

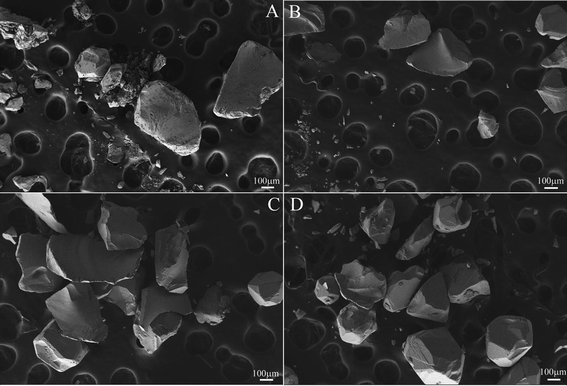

Supplement: Supplementary file 3 — Authors’ original file for figure 3 [file 12932_2014_10_MOESM3_ESM.gif]

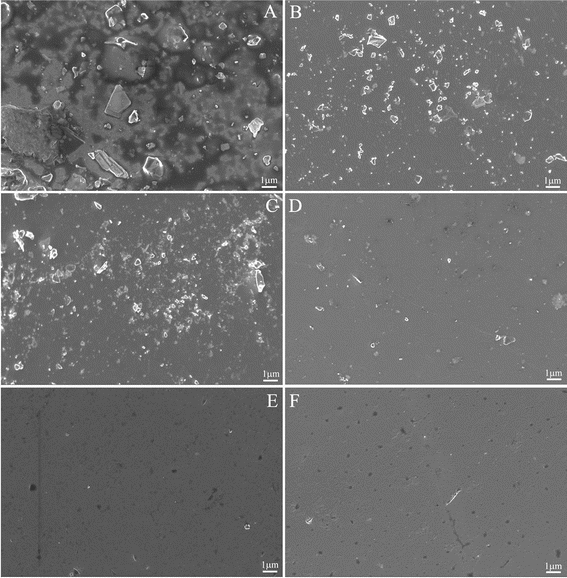

Supplement: Supplementary file 4 — Authors’ original file for figure 4 [file 12932_2014_10_MOESM4_ESM.gif]
